# Diel pattern of circadian clock and storage protein gene expression in leaves and during seed filling in cowpea (*Vigna unguiculata*)

**DOI:** 10.1186/s12870-018-1244-2

**Published:** 2018-02-14

**Authors:** Julia Weiss, Marta I. Terry, Marina Martos-Fuentes, Lisa Letourneux, Victoria Ruiz-Hernández, Juan A. Fernández, Marcos Egea-Cortines

**Affiliations:** 1 Genetics, ETSIA, Instituto de Biotecnología Vegetal, Universidad Politécnica de Cartagena, 30202 Cartagena, Spain; 2 Mapping Consulting, 26 Rue St Antoine du T, 31000 Toulouse, France; 3 Producción Vegetal, ETSIA, Instituto de Biotecnología Vegetal, Universidad Politécnica de Cartagena, 30202 Cartagena, Spain

**Keywords:** Storage proteins, Circadian rhythm, LHY, ELF3, TOC1, Legumine, Convicilin

## Abstract

**Background:**

Cowpea (*Vigna unguiculata*) is an important source of protein supply for animal and human nutrition. The major storage globulins VICILIN and LEGUMIN (LEG) are synthesized from several genes including *LEGA, LEGB, LEGJ* and *CVC* (CONVICILIN). The current hypothesis is that the plant circadian core clock genes are conserved in a wide array of species and that primary metabolism is to a large extent controlled by the plant circadian clock. Our aim was to investigate a possible link between gene expression of storage proteins and the circadian clock.

**Results:**

We identified cowpea orthologues of the core clock genes *VunLHY, VunTOC1, VunGI* and *VunELF3,* the protein storage genes *VunLEG, VunLEGJ,* and *VunCVC* as well as nine candidate reference genes used in RT-PCR. *ELONGATION FACTOR 1-A (ELF1A)* resulted the most suitable reference gene. The clock genes *VunELF3*, *VunGI, VunTOC1 and VunLHY* showed a rhythmic expression profile in leaves with a typical evening/night and morning/midday phased expression. The diel patterns were not completely robust and only *VungGI and VungELF3* retained a rhythmic pattern under free running conditions of darkness. Under field conditions, rhythmicity and phasing apparently faded during early pod and seed development and was regained in ripening pods for *VunTOC1* and *VunLHY*. Mature seeds showed a rhythmic expression of *VunGI* resembling leaf tissue under controlled growth chamber conditions. Comparing time windows during developmental stages we found that *VunCVC* and *VunLEG* were significantly down regulated during the night in mature pods as compared to intermediate ripe pods, while changes in seeds were non-significant due to high variance. The rhythmic expression under field conditions was lost under growth chamber conditions.

**Conclusions:**

The core clock gene network is conserved in cowpea leaves showing a robust diel expression pattern except *VunELF3* under growth chamber conditions. There appears to be a clock transcriptional reprogramming in pods and seeds compared to leaves. Storage protein deposition may be circadian regulated under field conditions but the strong environmental signals are not met under artificial growth conditions. Diel expression pattern in field conditions may result in better usage of energy for protein storage.

**Electronic supplementary material:**

The online version of this article (10.1186/s12870-018-1244-2) contains supplementary material, which is available to authorized users.

## Background

Cowpea (*Vigna unguiculata*) is an important food source of African origin and was introduced to the Indian subcontinent approximately 2000–3500 years ago [1]. It is used at all stages of growth from the green leaves, which are used like spinach, immature pods, green cowpeas or dry mature seeds [2]. Like other legumes and cereals, cowpea forms an important source of protein supply for human nutrition [3]. The accumulation of seed storage proteins in legumes occurs during the seed filling phases until desiccation. On average, legume seeds contain 17–30% protein (dry weight base) [4]. Protein content in cowpea varies between 14.8 and 23.6%, in Spanish landraces [5]. Globulins constitute with 55–58%, the major seed proteins in cowpea followed by albumins, basic glutelins, acid glutelins and prolamins [3, 6]. This compositional characteristic is stable in a nutritional survey of seed protein types in high-yielding cowpea cultivars [6].

Among the globulins, legumes mainly accumulate 7S vicilin-type globulins and 11S legumin-type globulins. The proportions of legumin (LEG) and vicilin (VIC) are genetically and environmentally determined in pea seeds and they are synthesized from at least 40 genes and at least 10 different genetic loci [7]. LEG and VIC share sequence identity both at the amino acid and nucleotide level, which hints to a common ancestor for these two storage proteins [8]. The *LEG* genes are arranged in two clusters for *LEGA* and *LEGB* [9]. Other genes codifying for minor B-type LEG polypeptides are *LEGJ* and *LEGK* in *Pisum sativum* L. (Gatehouse et al. [10]). A time series study of transcript profiles based on a *Lotus japonicus* gene expression atlas identified genes for *VIC*, *CVC* and *LEG* amongst the ten most highly expressed genes during legume seed maturation. Storage protein genes for *LEG* and *VIC* are also part of the pod-enhanced transcriptome [11].

Many plant biological activities show diurnal variation and the circadian clock acts as endogenous timer, coordinating and entraining plant activities in response to environmental cues such as light and temperature [12]. Biological activities under circadian control include those related to seasonal development such as flowering time, productivity, tuberization, and dormancy. Other biological processes controlled by the circadian clock are adaptation to cold or drought [13], pathogen resistance, stomatal movement, and scent production [14]. Genes related to primary metabolism, including RNA, proteins and carbohydrates, are expressed cyclically [15]. In Arabidopsis, 6% to 8% of all the open reading frames could be circadian regulated [16, 17]. Circadian regulation under light cycling involves 23% of the annotated genes in maize (Hayeset al. 2010) and 30–40% in rice and poplar (Filichkin et al. 2011). The patterns of transcription under circadian regulation may show distinct phasing, i.e. protein synthesis and cell cycle related processes have peaks between midnight and dawn, while those related to energy metabolism peak after dawn [18].

The circadian oscillator is well understood in *Arabidopsis thaliana* and contains a central circadian oscillator complex formed by the genes *CIRCADIAN CLOCK ASSOCIATED1/LATE ELONGATED HYPOCOTYL (CCA1/LHY).* These MYB genes act together with *TIMING OF CAB EXPRESSION1 (TOC1),* a PSEUDO-RESPONSE REGULATOR in a feedback regulating system, controlling its oscillation reciprocally [19, 20]. Interconnected with this core midday loop are a morning loop and an evening loop. The morning loop consists of three *PSEUDO RESPONSE REGULATOR (PRR)* genes *PRR9*, *PRR7* and *PRR5*. The protein products form a complex and inhibit the midday loop genes *CCA1* and *LHY* during the day. *CCA1* and *LHY* rise during late night and inhibit the evening loop consisting of *EARLY FLOWERING 3* (*ELF3*), *EARLY FLOWERING 4* (*ELF4*) and *LUX ARRHYTHMO (LUX)* as well as *GIGANTEA (GI)* and *ZEITLUPE (ZTL). ELF3, LUX,* and *ELF4* in turn inhibit the morning complex *PRR* genes in the early night, thus closing the negative feedback cycle [21].

The analysis of several other species, including CAM plants where photosynthesis is divided into a day and night phase, shows conservation of core clock genes but changes in their expression patterns [22, 23]. The genetic structure of the plant circadian clock has been analysed in *Solanaceae* where there are conserved gene duplications of some of the clock genes [24]. The genetic and transcriptional architecture of soybean leaves and seeds indicates somewhat conserved structure when compared to Arabidopsis [13, 25]. While *GmCCA1* and *GmLHY,* show conserved expression patterns, *GmTOC1* differs, indicating some basal modification [13]*.* Microarray expression profiling in developing soybean seeds shows that 1.8% of the mRNAs detected in seeds with predicted functions in protein synthesis, fatty acid metabolism, and photosynthesis are expressed in a circadian rhythm. Thus, circadian clock genes are probably controlling the gating of these processes in seed tissue. No information exists for the transcription of circadian clock genes in pods and in relation to storage protein accumulation.

Although the plant circadian clock is known with detail in Arabidopsis leaves and seedlings, there is increasing data showing that there are differences in the timing and genetic network structure in different plant tissues [26]. Mesophyll cells and vasculature have distinct circadian timings where the vasculature regulates the clock in other tissues [27]. The major differences in transcriptional structure between leaves and roots are apparently due to different light inputs and are responsible for the adaptation to complete dark in roots and day/night changes in leaves [28, 29].

In the present work, we identified the core clock genes and genes coding for storage globulins in cowpea by phylogenetic analysis. We analysed changes in gene expression in leaves, pods and seeds, both in 6 h intervals during 24 h for field grown samples and in 3 h intervals during 48 h in a growth chamber. We found a strong clock structure in leaves that faded away during early stages of pod development, resetting back in immature seeds. Furthermore, the genes coding for storage protein show a diel expression pattern, indicating a relationship between environmental inputs and protein synthesis.

## Results

### Phenotyping of pods and seeds

**Fig. 1:**
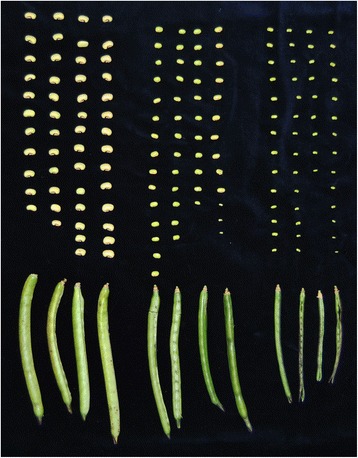
Pods of cowpea with their respective seeds used in the current study. From left to right completely grown pods and seeds before maturation started, intermediate pods and seeds and immature pods and seeds

In order to gain a more profound insight into the developmental patterns of cowpea seeds and pods, we characterized the growth in weight, length and width for 10 pods and 5 seeds per pod at 4, 7 and 15 days after anthesis of these generative tissues (Fig. 1).

**Fig. 2:**
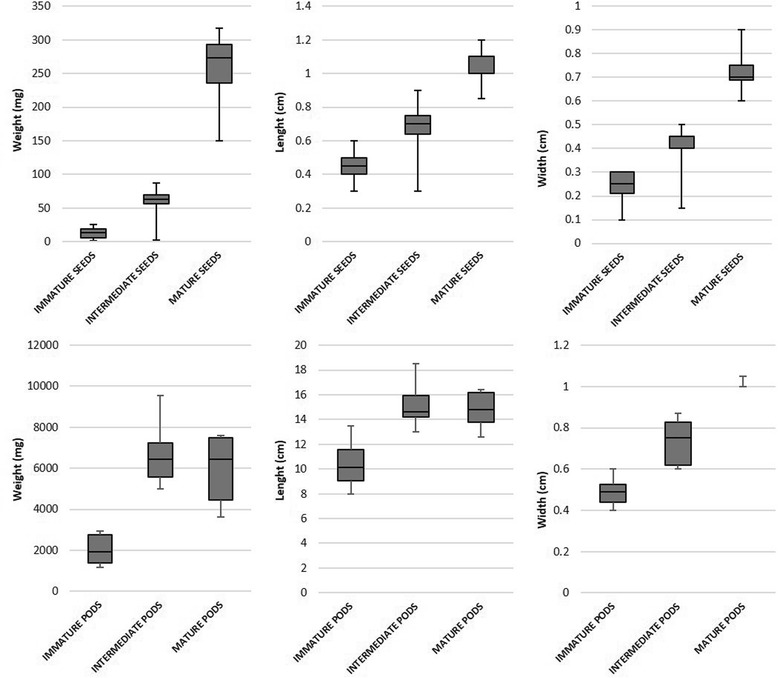
Boxplot of weight, length and width of seeds and pods in immature, intermediate and mature stage

Seed weight, length and width increased during the entire pod development, even so weight increases were more prominent during the second week after anthesis (Fig. 2). Differences were significant for all parameters both between immature and intermediate seeds as well as between intermediate and mature seeds (Wilcoxon test *p* = 0 for weight; ANOVA *p* = 0 for length and width). The lightest immature and the heaviest mature seeds showed a weight difference of 300 mg and length and width varied by a maximum of 1 cm.

Whole pod weight, including the seeds, increased up to 6000 mg between the immature and mature ripening stage (Fig. 2). Differences in pod weight were most prominent between immature and intermediate pods (ANOVA *p* = 0), while no significant differences were observed between intermediate and mature tissue (ANOVA *p* = 0.67). Some of the intermediate pods were heavier than some of the mature pods. Similar results were observed for pod length with significant differences between immature and mature (ANOVA *p* = 0) but not between intermediate and mature pods (ANOVA *p* = 0.9513766.

As already observed for weight, some intermediate pods were longer than the mature pods included in the analysis. We measured up to 8 cm of difference in length between the shortest and the longest pods, while pod width varied by 0.6 cm from the thinnest to the widest pod. Pod width increased continuously during development with significant differences both between immature and intermediate (Wilcoxon test *p* = 0.006) and between intermediate and mature pods (Wilcoxon test *p* = 0.002). Mature pods had an extremely homogeneous width (Fig. 2).

### Identification of genes for normalization, circadian clock genes and storage protein genes in cowpea

We obtained sequences for the normalization genes *Β-ACTIN (ACT), ACTIN 2/7 (ACT27), CYCLOPHYLIN (CYP), ELONGATION FACTOR 1-A (EF1A), ELONGATION FACTOR 1-B (EF1B), ALPHA TUBULIN (TUB4), BETA TUBULIN (TUB4), ASK-INTERACTING PROTEIN 16 (SKIP16)* and a *HYPOTHETICAL PROTEIN UNKNOWN* from soybean *(UKN2).* The clock genes were *VunLHY*, *VunTOC1*, *VunELF3* and *VunGI*, while the storage protein genes were *VunCVC, VunLEG* and *VunLEGJ*. We identified the selected genes by end-point PCR on genomic DNA. All primers gave single clear amplification products with the expected size, except for *TUB4* that showed an apparent size of 500 bp as compared to the expected 250 bp. This, however, was the result of amplifying a short intron present in the gene (Additional file 1: Figure S1). *VunVIC* was discarded from the analysis due to unspecific amplification products. Sequencing results of *VunVIC* also indicated a mixture of amplification products. We also tested the quality of the amplification in quantitative PCR to assess the melting profile of the PCR products, which gave single peaks at constant T_m_ for all tissues and developmental stages (Additional file 1: Figure S2). Sequence alignments with the identified *V. unguiculata* clones derived from Noble VuGEA database confirmed the correct identity of amplification products for all genes (Additional file 1: Figure S3).

Sequences encoding putative circadian clock genes in cowpea were identified through alignment of amino acid sequences of core circadian clock genes from Arabidopsis and several legumes including *Glycine max*. We identified potential soybean homologs of *GI*, *ELF3, TOC1* and *LHY*. In order to establish the putative orthology of the different transcripts identified, we performed a phylogenetic analysis of the cowpea genes.

**Fig. 3:**
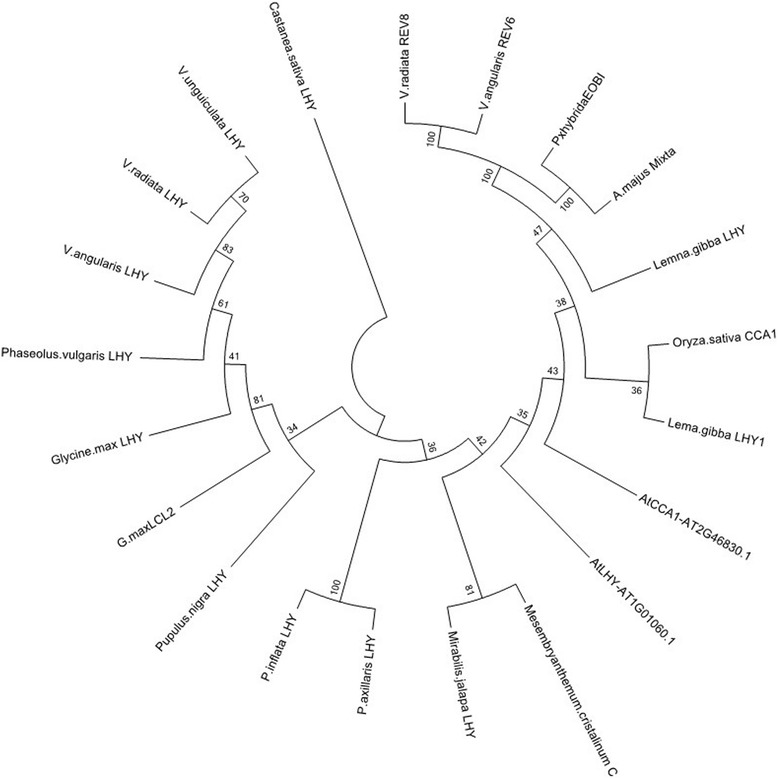
Phylogenetic tree of *LHY* related predicted proteins. The analysis involved 21 amino acid sequences. All positions containing gaps and missing data were eliminated. There was a total of 82 positions in the final dataset

The gene *VunLHY* was found as a single scaffold suggesting that, as previously reported for model legumes [30] and other species such as Petunia or *Solanum lycopersicum,* it is a single copy gene [24]. The *LHY* orthologs of the legumes *Phaseolus vulgaris*, *Glycine max*, *Vigna radiata*, *V. unguiculata* and *V. angularis* appeared in a phylogenetic reconstruction on a single clade comprising genes from *Castanea* and *Populus* (Fig. 3)*.* They were separated from a second major clade comprising the Arabidopsis *LHY*, the paralog *CCA1* and other *MYB* genes such as *MIXTA* from *Antirrhinum majus* [31] or *ENHANCER OF BENZENOID* from Petunia [32].

**Fig. 4:**
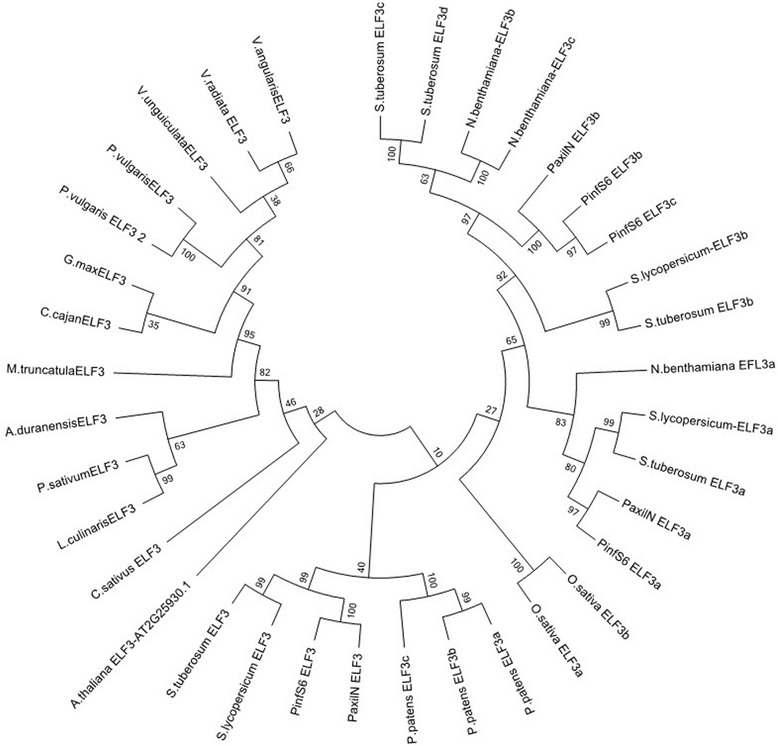
Phylogenetic tree of *ELF3* related predicted proteins. The analysis involved 36 amino acid sequences. All positions containing gaps and missing data were eliminated. There was a total of 105 positions in the final dataset

The gene *ELF3* is a single copy gene in Arabidopsis but is present in two to four copies in other species such as Petunia or *Physcomitrella* [24, 33]. Although *ELF3* had been previously reported as being absent from soybean [30], a recent ORFeome analysis under drought conditions identified a bona fide *Glycine max ELF3* gene [34]. The complete set of *ELF3* genes from legumes formed a distinct clade separated from a second one comprising the *ELF3* paralogs found in Solanaceae, *Arabidopsis*, *Oryza* and *Physcomitrella*. This suggests an early separation of this gene in legumes (Fig. 4).

**Fig. 5:**
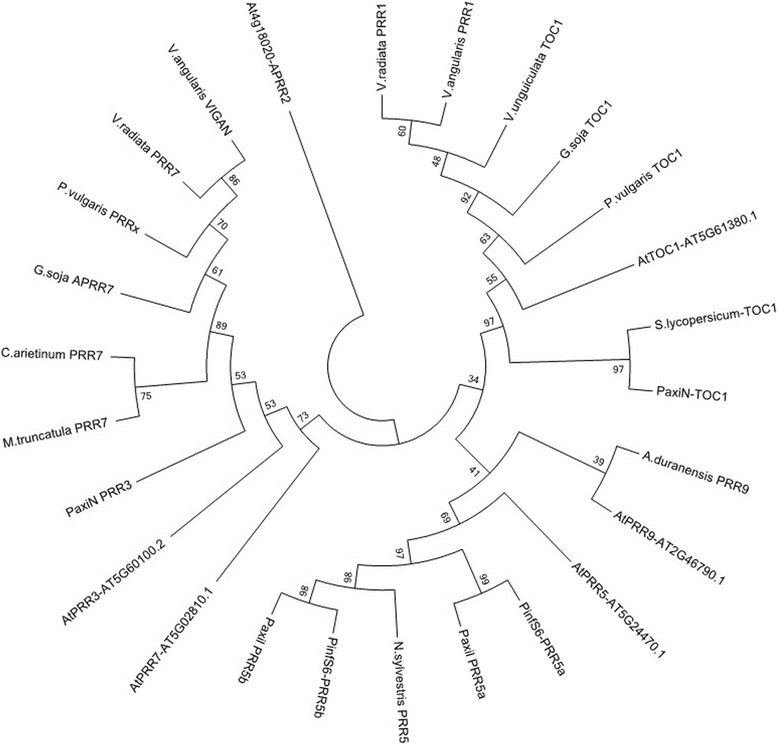
Phylogenetic tree of *TOC1* related predicted proteins. The analysis involved 26 amino acid sequences. All positions containing gaps and missing data were eliminated. There was a total of 84 positions in the final dataset

We analysed the phylogeny of *VunTOC1* and found that it formed a subclade with the rest of the *TOC1* genes (Fig. 5), and clearly separated from the *PRR9/5* and *PRR7/3*. In contrast to the tree structures found for *VunLHY* (Fig. 3) and *VunELF3* (Fig. 4), the *TOC1* orthologs of legumes were closer to *AtTOC1*, indicating a strong degree of conservation.

**Fig. 6:**
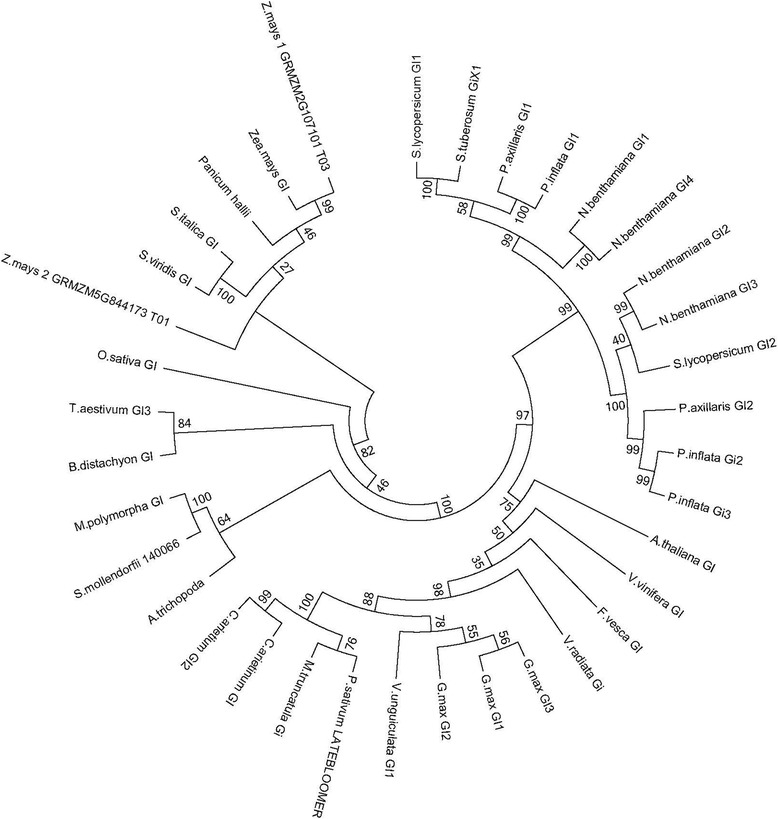
Phylogenetic tree of *GI* related predicted proteins. The analysis involved 42 amino acid sequences. All positions containing gaps and missing data were eliminated. There were a total of 640 positions in the final dataset

The *GIGANTEA* gene is a single copy gene in Arabidopsis and is found in one to four copies in *Solanaceae* and legumes [24, 35]. We found one scaffold and one EST (Vun_T01130.1_6). The ORF giving high homology to *GI* and orthologs was found in the − 3 frame suggesting that the aforementioned fragment had been annotated in the reverse orientation. The phylogenetic reconstruction of *VunGI* showed that, as previously found for *Solanaceae* and legumes [24], all *GI* orthologs clustered into three clades comprising monocots, dicots and basal angiosperms (Fig. 6). As expected *VunGI* clustered together with the rest of the legume genes used for this phylogenetic reconstruction. Within the different clades, duplicated genes such as those found in *Zea mays*, *Glycine* or *Nicotiana benthamiana*, showed differing levels of divergence, probably depending on the timing of whole genome duplications that occurred in these species.

### Data mining for stable reference genes

The identification of reference genes for normalization of quantitative PCR is a prerequisite for reliable gene-expression analysis [36]. We used *Β-ACTIN (ACT), ACTIN 2/7 (ACT27), CYCLOPHYLIN (CYP), ELONGATION FACTOR 1-A (EF1A), ELONGATION FACTOR 1-B (EF1B), ALPHA TUBULIN (TUB4), BETA TUBULIN (TUB4), ASK-INTERACTING PROTEIN 16 (SKIP16)* and a *HYPOTHETICAL PROTEIN UNKNOWN* from soybean *(UKN2).* There are a number of programs differing in the mathematical solution to identify stable genes including geNorm [37], Normfinder [38], Bestkeeper [39] and the comparative delta Ct methods [40]. Table 1 shows the ranking of reference genes for the different analysis software and as result of the pooled PCR analysis software. Table 2 gives the Geomean of ranking values of the candidate reference genes based on the geometric mean of the weights of every gene calculated by each program [41]. *ELONGATION FACTOR 1-A (EF1A)* was found to be the most suitable reference gene while *BETA TUBULIN (TUB4)* was the least suitable gene.

**Table 1:** Ranking of normalisation genes for cowpea transcriptomic analysis based on Rank-Aggreg

Method	1	2	3	4	5	6	7
Delta CT	EF1A	Tua4	Act	Cyp	Skip16	Act27	Tub4
BestKeeper	Act	Tua4	EF1A	Skip16	Cyp	Act27	Tub4
Normfinder	EF1A	Tua4	Skip16	Act	Cyp	Act27	Tub4
Genorm	AF1A/Tua4		Skip16	Act	Cyp	Act27	Tub4
Recommended comprehensive ranking	EF1A	Tua4	Act	Skip16	Cyp	Act27	Tub4

**Table 2:** Geomean of ranking values of the candidate reference genes

Gene	Geomean of ranking values
EF1A	1.32
Tua4	1.68
Act	2.63
Skip 16	3.66
Cyp	64.73
Act27	6.00
Tub4	7.00

### Circadian expression of the circadian clock genes *VunGI, VunELF3, VunTOC1, and VunLHY* in leaves

In order to determine the expression pattern of the clock genes in leaves, we grew plants in the field and in the greenhouse with a natural photoperiod of 15.5 h of light and 8.5 of dark. Plants were transferred to growth chambers and after acclimation for 3 days at a regime of 12:12 LD, they were subject to sampling for 2 days at 12:12 LD and transferred to continuous dark (DD) for 24 h.

**Fig. 7:**
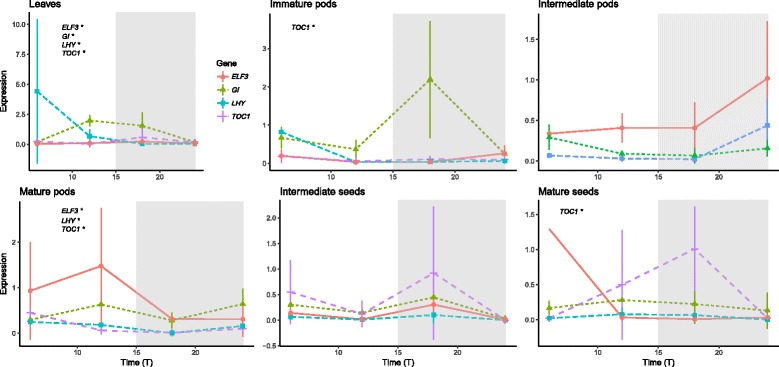
Expression of the circadian clock genes *VunGI, VunELF3*, *VunTOC1* and *VunLHY* in cowpea leaves, pods and seeds sampled under field conditions. Expression represents the normalized expression NE according to the formula (NE) = 2^-(Ct experimental – Ctn). Collection points represent Time 6, 12, 18 and 24 h after dawn. Four samples were analyzed for each time point and error bars indicate the standard deviation. A significant rhythm in the time series according to JTK_CYCLE is indicated with an asterisk

Under field conditions *VunGI, VunELF3*, *VunTOC1* and *VunLHY* showed a significant rhythm during a time series of 24 h (Fig. 7; Table 3). An evening/night-phased expression was observed for *VunELF3* and *VunGI* with the highest expression between 12 and 18 h after dawn and the lowest expression during morning and midday. We observed the highest peak phase expression amplitude for *VunLHY*, followed by *VunELF3* and *VunTOC1.*

**Table 3:** Statistical analysis of gene expression data

Field conditons	Leaves				Intermediate pods				Immature pods			
	Pval	Per	Phase	Amp	Pval	Per	Phase	Amp	Pval	Per	Phase	Amp
ELF3	0.00	18	0	0.75	1	18	15	0.15	1	18	0	0.45
GI	0.00	24	15	1.06	1	24	0	0.05	0.08	18	3	0.39
LHY	0.00	24	9	0.80	0.06	24	6	0.09	0.14	18	6	0.52
TOC1	0.00	18	3	0.12	0.00	24	3	0.15	1	18	3	0.04
LEG	–	–	–	–	0.03	24	9	0.14	0.00	24	0	0.06
LEGJ	–	–	–	–	0.12	18	3	0.09	0.52	24	9	0.01
CVC	–	–	–	–	1	18	12	0.05	0.14	18	12	0.01
	Mature pods				Intermediate sedes				Mature seeds			
	Pval	Per	Phase	Amp	Pval	Per	Phase	Amp	Pval	Per	Phase	Amp
ELF3	0.01	18	9	0.34	0.08	18	3	0.76	0.07	24	21	0.13
GI	0.10	18	12	0.18	0.50	18	3	0.10	0.09	24	12	0.05
LHY	0.00	24	6	0.08	1.00	18	3	0.01	0.30	24	15	0.01
TOC1	0.00	24	6	0.11	0.21	18	3	0.25	0.04	24	18	0.24
LEG	0.58	24	12	0.08	0.25	18	0	0.16	0.55	24	0	0.04
LEGJ	0.00	18	6	0.14	0.14	18	12	4.13	0.00	24	9	33.33
CVC	0.00	24	6	0.04	1.00	18	9	5.17	0.04	24	9	18.54
12LD (48 h)	Leaves				Mature pods				Mature seeds			
	Pval	Per	Phase	Amp	Pval	Per	Phase	Amp	Pval	Per	Phase	Amp
ELF3	0.07	27	24	1.21	–	–	–	–	–	–	–	–
GI	0.00	21	10.50	3.03	1	21	4.50	0.26	0.00	24	10.5	0.05
LHY	0.00	24	3	3.79	1	27	4.50	0.26	0.50	0	0	0
TOC1	0.00	24	18	0.54	1	21	1.50	0.47	1	21	13.50	0.00
LEG	–	–	–	–	1	27	3	0.00	1	24	15	0.00
LEGJ	–	–	–	–	1	27	7.50	0.07	0.13	27	24	18.90
CVC	–	–	–	–	1	27	4.50	0.16	0.07	27	28	14.22
12DD (24 h)	Leaves				Mature pods				Mature seeds			
	Pval	Per	Phase	Amp	Pval	Per	Phase	Amp	Pval	Per	Phase	Amp
ELF3	0.00	24	9	16.63	–	–	–	–	–	–	–	–
GI	0.00	21	10.50	3.93	0.70	24	21	1.08	1	24	6	0.32
LHY	0.18	24	3	1	0.01	24	22.50	0.43	0.37	24	3	0.04
TOC1	0.51	24	15	1.12	1	21	6	1.86	1	24	9	0.23
LEG	–	–	–	–	1	21	0	0.12	1	21	1.50	0.00
LEGJ	–	–	–	–	0.24	24	3	0.65	0.81	21	15	6.46
CVC	–	–	–	–	0.30	24	3	0.46	1	21	15	10.69

*P* value (Pval, significative if *P* < 0.05), period (Per), adjusted phase (Phases given by JTK_CYCLE and Lomb-Scargle need to be adjusted with their predicted period) and amplitude (Amp). Period is defined as the time between two consecutive peaks, phase is considered as the time point with the peak, amplitude is the difference between the peak (or minimum) and the mean value of the wave

**Fig. 8:**
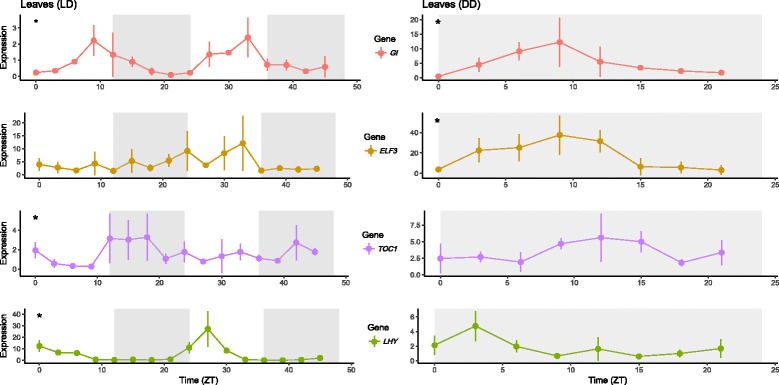
Expression of the circadian clock genes *VunGI, VunELF3*, *VunTOC1* and *VunLHY* in cowpea leaves sampled under growth chamber conditions. Expression represents the normalized expression NE according to the formula (NE) = 2^-(Ct experimental – Ctn). Samples were collected in 3 h intervals during 48 h (LD) and during 24 h under free-running condition. Three samples were analyzed for each time point and error bars indicate the standard deviation. A significant rhythm in the time series according to JTK_CYCLE is indicated with an asterisk

Under LD conditions, *VunGI, VunTOC1* and *VunLHY,* but not *VunELF3,* showed a significant rhythm (Fig. 8, Table 3). The expression of *VunGI* was highest towards the end of the light period with a peak phase at T9 and was lowest at dawn (T0). Under free-running conditions of continuous darkness (DD), *VunGI* showed a significant rhythmicity with peak expression at T10.5 of subjective time, indicating a robust circadian rhythmicity for this gene, although oscillation period was shortened to 21 h (Fig. 8 Table 3). Even though we did not find a significant oscillation of *VunELF3* during 48 h under LD conditions (Fig. 8; Table 3), this gene shows a significant rhythm under DD conditions. The expression level of *VunTOC1* increased towards the end of light period under LD conditions with peak phase at T18 (Fig. 8; Table 3). Although a similar expression pattern was observed under DD conditions, the pattern was not significant. *VunLHY* expression showed a peak phase at T3 under LD conditions (Fig. 8; Table 3). Similar to *VunTOC1*, the expression pattern of *VunLHY* was conserved under DD conditions, but the identified pattern was not significantly rhythmic.

### Diel expression of the circadian clock genes *VunGI, VunELF3, VunTOC1, and VunLHY* in pod and seed tissue

We performed a detailed expression analysis of the circadian genes, found to be rhythmic in leaves (Fig. 7; Table 3) in seeds and pods, in order to compare the circadian gene network among tissues and developmental stages.

Under field conditions, *VunTOC1* was the only rhythmic gene in immature pods. As development advanced to intermediate pods, rhythmic expression had been lost and showed very low expression levels (0.022–0.4) (Fig. 7 Table 3). Rhythmic expression was regained in mature pods for *VunELF3*, *VunLHY* and *VunTOC1*. The phase of the three genes was different from leaves changing from 0 to 9 for *VunELF3*, 9 to 6 for *VunLHY* and 3 to 6 for *VunTOC1* (Table 3).

Similar to pods, we could not find significant rhythms for any of the circadian clock genes in intermediate seeds. In mature seeds, only *VunTOC1* showed a significant rhythm.

Performing a comparative analysis of gene expression at different times of the day during development (Table 4) we found that the gene *VunTOC1* was significantly down regulated in immature versus mature pods at T18 (17.57 fold *p* = 0.03) and intermediate versus mature pods at T12 (3.83 fold *p* = 0.002).

**Table 4:** Comparative analysis of gene expression at different times of the day during development of different tissues. Differences

			Tissue											
			Immature pods vs mature pods			Immature pods vs intermediate pods			Intermediate pods vs mature pods			Intermediate seeds vs mature seeds		
	Gene	Time	*p* -Value	Expression factor	SE(±)	*p* -Value	Expression factor	SE(iJ	*p* -Value	Expression factor	SE(±J	*p*-Value	Expression factor	SE(±)
Protein storage genes	*VunCvc*	T6	0.12	29.65	3.72	0.35	8.32	3.17	0.96	3.57	1.33	0.48	137.41	5.12
		T12	0.02^*^	(−)16.99	2.29	0.97	1.25	0.14	0.06	(−)21.23	1.98	0.10	(−)1.56	0,57
		T18	0 30	(−)4.30	1.00	0.20	4.24	0.94	0.03^*^	(−)21.69	1.63	0.52	32.03	5.12
		T24	0.10	(−)3.77	1.07	0.83	(−)1.48	0.18	0.84	(−)2.70	0.29	0.99	(−)1.98	0,36
	*Vunleg*	T6	0.47	(−)4.71	0.33	0.46	3.97	1.82	0.02^*^	(−)18.68	1.72	0.48	2.69	3.86
		T12	0.24	(−)4.74	0.67	0.95	1.77	0.91	0.73	2.43	1.27	0.49	(−)1.85	1.46
		T18	0.70	(−) l.36	0.18	0.60	(−)2.15	0.33	0.77	1.61	0.97	0.51	(−)1.40	1.19
		T24	0.92	(−)8.77	1.60	0.63	(−)2.43	0.71	0.82	(−)3.54	0.26	0.96	(−)2.08	0,88
	*VunLegJ*	T6	0.17	7.15	1.61	0.79	2.91	1.18	0.93	2.49	0.50	0.52	74.83	3.59
		T12	0.30	(−)11.12	1.80	0.92	(−)3.96	1.13	0.54	(−)2.81	0.38	0.86	(−)8.74	0.59
		T18	0.43	(−)2.06	0.47	0.15	15.87	2.06	0.51	(−)2.72	0.82	0.86	3.50	3.05
		T24	0.97	2.15	0.95	0.93	1.71	0.38	0.92	1.26	0.75	0.95	(−)1.77	1.09
Clock genes	*VunGI*	T6	0.82	(−)1.65	0.27	0.72	(−)3.23	0.21	0.91	1.96	1.02	0.10	(−)5.20	0,85
		T12	0.99	(−)1.21	0.31	0.57	(−)7.57	0.92	0.31	6.23	2.84	0.88	3.24	1.37
		T18	0.10	(−)6.80	1.85	0.69	(−)3.03	0.42	0.86	(−)2.24	0.13	0.86	51.12	3.17
		T24	0.08	3.50	1.07	0.80	1.58	0.19	0.40	2.21	1.34	0.99	6.35	1.23
	*VunTOC*	T6	0.42	2.67	1.72	0.61	(−)2.11	0.28	0.40	5.65	1 98	0.98	(−)11.37	1.37
		T12	0.99	1.60	1.02	0.97	(−)2.40	0.09	0.002 ^**^	3.83	1.43	0.60	10.09	2.00
		T18	0.03^*^	(−)17.57	2.22	0.65	2.09	1.52	0.56	(−)36.67	2.66	0.99	6.35	1.83
		T24	0.88	(−)1.26	0.19	0.19	3.75	0.86	0.05	(−)2.97	0.08	0.83	5.97	0.58
	*VunlHY*	T6	0.43	(−)4.27	0.44	0.33	(−)3.15	0.19	0.85	1.32	0.50	1.00	(−)1.07	0,88
		T12	0.84	4.14	2.08	0.75	3.15	1.66	0.10	1.31	0.91	0.06	4.48	1.13
		T18	0.07	(−)8.28	1.93	0.99	(−)1.36	0.45	0.89	(−)6.11	1.10	0.88	10.39	1.49
		T24	0.37	3.04	1.77	0.68	2.75	11.80	0.96	1.10	0.07	0.99	1.80	1.47
	*VunElf3*	T6	0.49	(−)2.78	0,73	0.001^**^	(−)3.98	0.33	0.51	1.42	8.89	0.001 ^**^	105.26	7.68
		T12	0.51	6.74	7.90	0.47	(−)1.12	2.86	0.001 ^**^	17.79	5.11	0.49	12.07	4.18
		T18	0.52	4.92	3.20	0.51	2.64	1.62	0.53	5.49	2.83	0.51	(−)23.89	1.78
		T24	0.001^**^	40.62	1.58	0.52	33.35	1.28	0.001^**^	1354.72	1.85	0.51	5.71	13.36

Significance level: * = *p-*Value≤0.05 and ** = *p-*Value ≤0.01

We studied the expression of *VunGI*, *VunTOC1* and *VunLHY* in mature pods and seeds under controlled conditions. *VungGI* retained a rhythmic expression in mature seeds under LD but the rest of the genes lost the rhythmic expression pattern (Figs. 10 and 11 Table 3).

### Expression profile of storage protein genes in seeds and pods during development

**Fig. 9:**
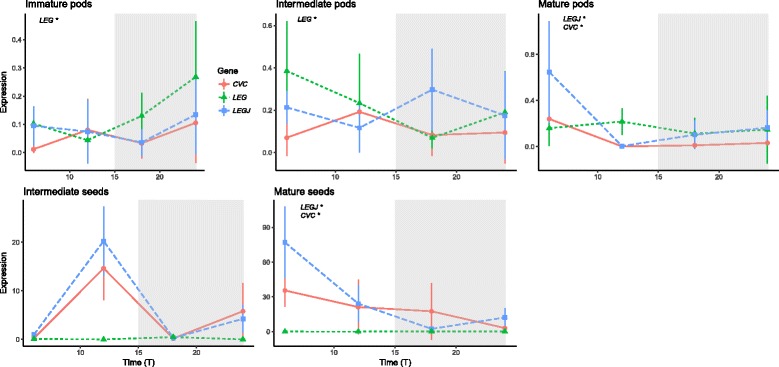
Expression of the protein storage genes *VunCVC*, *VunLEG* and *VunLEGJ* in cowpea pods and seeds under field conditions. Expression represents the normalized expression NE according to the formula (NE) = 2^-(Ct experimental – Ctn). Collection points represent Time 6, 12, 18 and 24 h after dawn. Four samples were analyzed for each time point and error bars indicate the standard deviation. A significant rhythm in the time series according to JTK_CYCLE is indicated with an asterisk

We analyzed the relative expression of storage proteins *VunCVC, VunLEG* and *VunLEGJ* in leaves, pods and seeds in 6 h intervals under field conditions (Fig. 9). We did not detect expression of *VunCVC, VunLEG* and *VunLEGJ* in leaves (data not shown). The expression pattern of *VunLEG* was rhythmic in intermediate and immature pods while *VunLEGJ* and *VunCVC* showed significant changes in expression patter in mature pods.

Storage genes were not rhythmic in intermediate seeds but *VunLEGJ* and *VunCVC* had a clear rhythmic expression during the mature seed stages (Fig. 9).

**Fig. 10:**
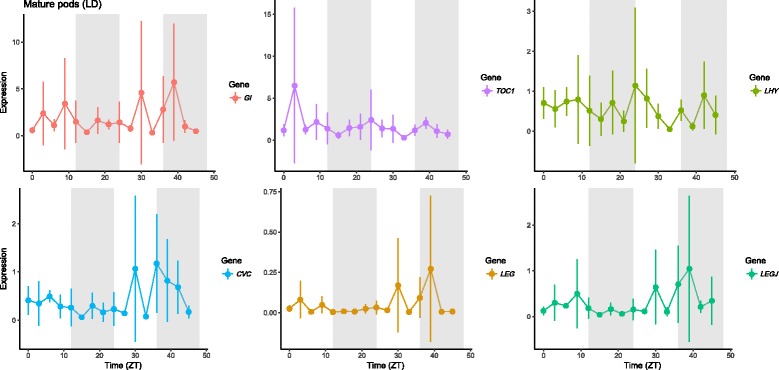
Expression of the circadian clock genes and protein storage genes in mature cowpea pods sampled under growth chamber conditions. Expression represents the normalized expression NE according to the formula (NE) = 2^-(Ct experimental – Ctn). Samples were collected in 3 h intervals during 48 h (LD). Three samples were analyzed for each time point and error bars indicate the standard deviation

**Fig. 11:**
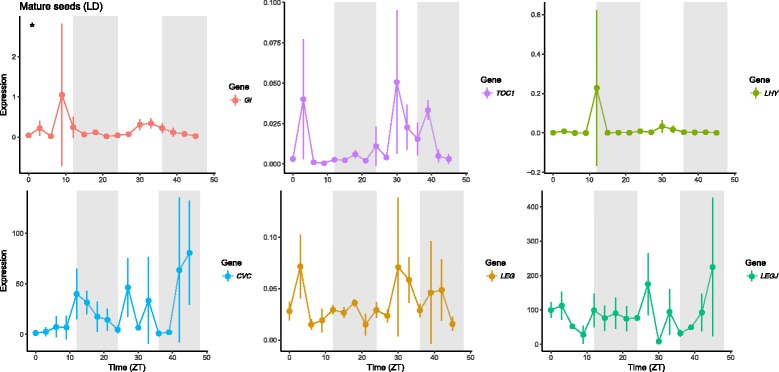
Expression of the circadian clock genes and protein storage genes in mature cowpea seeds sampled under growth chamber conditions. Expression represents the normalized expression NE according to the formula (NE) = 2^-(Ct experimental – Ctn). Samples were collected in 3 h intervals during 48 h (LD). Three samples were analyzed for each time point and error bars indicate the standard deviation

We analysed the expression under controlled conditions in a growth chamber under LD conditions, and as previously found for the clock genes, the strong rhythmic expressions were lost both in mature pods and mature seeds (Figs. 10 and 11, Table 3), the two tissues where a rhythmic expression had been found in field conditions (Fig. 9).

We compared expression levels during development of seeds and pods of field samples considering the different sampling times (Table 4). Several protein storage genes were found to be significantly downregulated at certain time points in ripening versus mature tissue, including *VunCVC* in immature versus mature pods at T12 (16.99 fold *p* = 0.02), and in intermediate versus mature pods at T18 (21.69 fold *p* = 0.04) as well as *VunLEG* in intermediate versus mature pods at T6 (18.68 *p* = 0.02).

## Discussion

### Pod and seed growth

The development of pods and seeds occurs in a rapid way in many legume plants such as pea, Medicago or soybean [42–44]. In this work, we have performed a detailed characterisation of the developmental pattern of cowpea seeds and pods at 4, 7 and 15 days after anthesis. Our results show that indeed growth occurred during a short period of roughly 2 weeks. Whilst seed weight, length and width increased during the entire pod development, increases were more prominent during the second week after anthesis. Also, whole pod weight, including the seeds, increased until pod maturity, but most notably during the first week after anthesis. Compared to seeds we found that pod weight and length is more variable, as some of the intermediate pods were heavier and longer than some of the mature pods. Our results suggest a variance in achieving maximal length that maybe up to a week. The last stages of cowpea seed development show a strong decrease in seed moisture to 20% or less [45], thus explaining the loss in weight of some mature pods. On the other hand, mature pods had an extremely homogeneous width. Our data indicate that growth of cowpea pods, including the seeds, both in weight and length, occurs during the first week after pollination. Thereafter, pods further expand only in width.

The fact that whole pod weight increases notably during the first week after anthesis but seed weight during the second week, indicates, that initial pod growth is the result of pod tissue growth with a second week where tissue width is taking over as a result of seed filling. Altogether, our experiments display a consistent pattern, expected for the short length cowpea belonging to the *Vigna unguiculata var. ungiculata* group [46].

### Identification of genes for normalization, circadian clock genes and storage protein genes in cowpea

We successfully identified gene homologues for normalisation, circadian clock and storage proteins from *V. unguiculata*. As the identified sequences were designed to be used in qPCR assays, a careful analysis of the resulting PCR products and their sequences is required in order to rule out unspecific amplifications. Based on end-point PCR with genomic DNA, dissociation curve analysis and sequencing alignments with *V. unguiculata* clones derived from Noble VuGEA database, all primers used in this study gave single clear amplification products identical to the corresponding Noble VuGEA clones, confirming the correct identity of the cowpea genes. We further performed a phylogenetic analysis of the clock genes *GI*, *ELF3, TOC1* and *LHY* to obtain a profound view of the genetic structure of clock genes in cowpea. The phylogenetic structure of the clock genes revealed what appears to be a genetic structure of a diploid plant, confirming the current data on cowpea [47]. Clock genes are thought to be preferentially retained after genome duplications [48]. Indeed clock genes duplicated in soybean such as *GmGI* [49] are found as single copy genes in cowpea indicating that the set of clock genes is similar to the standard structure of Arabidopsis except for *LHY* where we found a single copy gene.

### Data mining for stable reference genes

We used the programs geNorm, Normfinder, Bestkeeper and the comparative delta Ct methods in order to obtain a ranking of reference genes for normalization of quantitative PCR gene expression analysis. Ideally, expression of a reference gene should be independent of the morphogenetic process in order to validate transcriptomic changes of target genes during plant development. *ELONGATION FACTOR 1-A (EF1A)* was found to be the most suitable reference gene while *BETA TUBULIN (TUB4)* was the least suitable gene. *EF1A* was also shown to be a suitable reference gene for potato during biotic and abiotic stress conditions [50] and for Petunia over a wide range of developmental stages [36], varieties, mRNA extraction and qPCR procedures. *VuEF1A* was further used for normalisation of transcript levels of clock genes and storage protein related genes in this study.

### Circadian expression of the circadian clock genes *VunGI, VunELF3, VunTOC1, and VunLHY* in leaves

A significant rhythm in the expression pattern was observed for the four clock genes *VunGI, VunELF3*, *VunTOC1* and *VunLHY* under field conditions, and patterns of rhythmicity coincided with those reported for other species except in case of *VunTOC1*. A typical evening/night-phased expression was observed for *VunELF3* and *VunGI* with the highest expression between 12 and 18 h after dawn and the lowest expression during morning and midday, while we did not observe significance for this type of rhythmicity in the expression of *VunTOC1* that rather showed a significant peak towards midday. Nevertheless, the relative expression amplitude over 24 h resembled those found in soybean leaves [13]. In contrast, *VunLHY* expression was comparable to the typical morning peak observed over a wide range of tissues in Arabidopsis [19]. This may be due to the fact that *CCA1/LHY* is regulated through a negative feedback loop not only by *TOC1*, but together with other genes, including *GI, ELF3, ELF4* and *LUX* [12].

We detected two divergences in clock gene rhythmicity during 48 h under LD conditions compared to field results. First, *VunELF3* did not oscillate significantly, and surprisingly a significant rhythm was seen under DD conditions. Second, the expression level of *VunTOC1* increased towards the end of the light period and not towards midday as in field samples. However, it can be generally concluded that the expression pattern of the cowpea clock genes was basically conserved in leaves when compared to other species [13, 19, 51]. These results are similar to the typical morning and evening oscillator peaks observed over a wide range of tissues in Arabidopsis [19] as well as in legumes [13]. Under free running conditions, peak phase and 24 period were maintained, but amplitude was dampened and as a result, rhythmicity was not significant. The negative feedback regulation of *CCA1/LHY* by *TOC1* together with other genes, including *GI, ELF3, ELF4* and *LUX* [12], leads to the coincidence of lowest expression of *VunLHY* with highest of *VunTOC1* and *VunGI* and vice versa*.* Similar to observations in soybean leaves, were relative expression amplitude at peak phase was highest for *GmLHY* followed by *GmGI*, *GmPRR* genes, including *GmTOC1*, and *GmELF4* [13], we observed the highest expression amplitude for *VunLHY* and the lowest for *VunTOC1*, both under LD48 and field conditions. Under free-running conditions, amplitudes of *VunGI* and *VunTOC1* were higher and *VunLHY* lower compared to LD48, which may be related to the differing temperature conditions [52].

### Diel expression of the circadian clock genes *VunGI, VunELF3, VunTOC1, and VunLHY* in pod and seed tissue

The analysis of the circadian gene network in generative cowpea tissues during development showed again that clock gene rhythmicity diverged between field and LD conditions. Field samples of pods and seeds showed rhythmic oscillation of several clock genes primarily at mature stage with phasing different from leaves, indicating a clock resetting in the mature generative tissue. However, under LD conditions, most of the genes had lost the rhythmic expression pattern except for *VungGI* in mature seeds under LD.

The differences in clock genes expression during development became especially clear after performing a comparative analysis of gene expression at different times of the day. This analysis showed that the gene *VunTOC1* was significantly downregulated at specific hours of the day in earlier phases of development compared to mature pods. This suggests that sampling during the day is relevant for comparing gene expression patterns among tissues.

Our results show that the circadian oscillation of the clock genes is strong in leaves and resilient to environmental inputs. Lateral reproductive organs go through a period (immature pods, intermediate seeds) where diel expression patterns of clock genes cannot be found. A different expression pattern gets established again late in development. The reproductive organ shows a diel pattern in field conditions but disappears under growth chamber conditions, indicating that it is probably caused by the strong environmental inputs (light and temperature changes) found in the field. Altogether we can conclude that organ-specific clock transcriptional setups may undergo reprogramming via shutting down and restarting expression albeit with newly defined diel patterns.

### Expression profile of storage protein genes in seeds and pods during development

The storage protein genes *VunCVC, VunLEG* and *VunLEGJ* showed a rhythmic expression pattern under field conditions that depended on tissue type and developmental stage. *VunLEGJ* and *VunCVC* showed significant changes in expression pattern only in mature seeds and pods, while *VunLEG* significantly oscillated only in immature and intermediate pods. A predominant rhythmic oscillation primarily at mature stage in seeds was also found for the circadian clock genes, indicating a transcriptional reprogramming of both clock and some genes coding for storage proteins.

As previously found for the clock genes, the strong rhythmic expressions of storage protein genes were lost both in mature pods and mature seeds under LD conditions. These results indicate that under field conditions, the environmental inputs are the ones sustaining diel expression patterns and these expression patterns are more fragile in the generative tissues. There are several metabolic pathways that show circadian expression such as scent or anthocyanin synthesis [53, 54]. Under free running conditions, dampening i.e. a decrease in cycling amplitude appears to be gene and organ specific. Our results show that this is the case not only for the clock genes but also for the protein storage genes that appear to be driven to a large extent by environmental inputs in field conditions.

As already shown for clock genes, the expression of several protein storage genes differed significantly depending on the specific time point of sampling and the developmental stage, showing a general tendency of downregulation in ripening versus mature tissue. These results again show that sampling times can play a key role when assessing gene expression in cowpea seeds.

## Conclusion

Gene expression analysis by RT-PCR requires appropriate reference genes with stable expression in a wide range of tissue types, developmental stages and sampling times. We identified *VuELF1A* as the most appropriate gene for transcription analysis in cowpea. Using this reference gene, we found that the storage protein genes *VunLEG, VunLEGJ and VunCVC* are expressed during all ripening stages of pods and seeds. Maximal expression was found in mature seeds followed by intermediately ripe seeds and pods. Differences in average gene expression during ripening were especially pronounced in seeds and to a lesser extent in pods, even so differences were mostly non-significant except for particular storage protein genes at particular time points. Storage protein levels at maturity are the accumulated result of gene expression during the entire organ development and expression changes are therefore difficult to relate to absolute protein levels. Nevertheless, our data hint to a parallelism between storage protein content and gene expression level, which are both lower in pods than seeds and highest in mature seeds. The core clock genes *VunGI, VunTOC1* and *VunLHY* showed a stable circadian oscillation with typical peak phases in cowpea leaves. Of these genes, only *VunGI* seemed to conserve a rhythmic expression in mature seeds. Storage protein gene expression showed daily changes. Even though changes in expression of *VunLEGJ* and *VunCVC* in mature seeds sampled under field conditions show rhythmicity (Fig. 10), these changes did not proof to follow a robust circadian oscillation as evaluated under controlled environmental conditions or free running conditions. While circadian clock genes tend to be robust in circadian expression in leaves, the degree of robustness shown by different clock regulated biological processes seems to be variable. For instance starch degradation or anthocyanin synthesis are labile [53, 55], while root extension is resilient [56]. Legume seed protein gene expression is metabolically regulated through changes in osmotic pressure or soluble sugar concentrations [57] and these factors may contribute to diel changes observed here. Our results emphasize on the importance of coordinating sampling time for comparative expression analysis of storage protein genes, for example when evaluating levels of protein storage gene expression as marker tor protein content in cowpea varieties. The strong diel pattern found in field conditions indicate a possible gating of metabolic aspects related to improving carbohydrates and nitrogen from leaves to the grains. Thus, the differences found in the clock between a source and a sink organ appear biologically meaningful.

## Methods

### Plant material, phenotyping and sampling

Plant material was sampled both under controlled conditions in a growth chamber and under field conditions. For the latter, the IT97K-499-35 breeding line of cowpea was grown under field conditions at the “Tomás Ferro” Experimental Agro-Food Station, Technical University of Cartagena located in southeast of Murcia region, Campo de Cartagena, Spain. Average temperatures during sampling time were 29.1 °C. Leaves and pods at different stages of development were sampled at 6:45 am, 12:45 pm, 6:45 pm, 00:45 am These times corresponded in July to subjective time of T0, T6, T12 and T18 considering T0 as (dawn). Time of sunset was at 9:31 pm. Expression of reference genes was analysed at T6 and T18, clock genes and protein storage genes at T0, T6, T12 and T18. Leaves were harvested when first pods matured. Developmental stages of pods were categorized based on the phenotyping of whole pods containing seeds and seeds only. Phenotyping included measurements of weight, length and width of 10 pods and 5 seeds per pod for three ripening stages: immature, intermediate and mature (Fig. 1). These stages corresponded to 4 days, 7 days and 15 days after anthesis. Gene expression analysis was performed using four independent samples of leaves as well as inmature, intermediate and mature pods and intermediate and mature seeds at each time point. The seeds were harvested from different pods. For plant sampling under controlled conditions, the IT97K-499-35 breeding line was first grown under greenhouse conditions in 5 L pots and transferred to a growth chamber for acclimatisation during 3 days prior to sampling under a photoperiod of 12 h/12 h of light/dark and 27 °C/16 °C temperature. The stages of tissue sampling correspond to those described above. Tissue sampling was performed during 48 h of light/dark cycle in 3 h intervals as well as under free running conditions of complete darkness during 24 h at 16 °C. Gene expression analysis was performed from leaves and mature pods and seeds using three independent samples from three plants.

### Identification of genes for normalization, circadian clock genes and storage protein genes in cowpea

We identified candidate reference genes from legumes using the gene expression atlas from *Medicago truncatula* [58] and a set of genes found suitable for normalization in soybean [59]. We used the accession numbers to identify cowpea genomic sequences by BLAST (harvest-web.org). Scaffolds were retrieved and using legume translated mRNAs, we identified putative mRNAs from cowpea using Genewise [60] and Noble VuGEA (Additional file 1: Table S1). The genes used were *Β-ACTIN (ACT), ACTIN 2/7 (ACT27), CYCLOPHYLIN (CYP), ELONGATION FACTOR 1-A (EF1A), ELONGATION FACTOR 1-B (EF1B), ALPHA TUBULIN (TUA4), BETA TUBULIN (TUB4), ASK-INTERACTING PROTEIN 16 (SKIP16)* and a *HYPOTHETICAL UNKNOWN PROTEIN* from soybean *(UKN2).* The genes related to protein storage accumulation were *LEGUMIN (LEG), LEGUMINJ (LEGJ) and COVICILIN (CVC).* Circadian clock related genes were *GIGANTEA (GI), TIMING OF CAB EXPRESSION1 (TOC1), LATE ELONGATED HYPOCOTYL (LHY),* and *EARLY FLOWERING 3(ELF3).* Primers were designed using the software PCRefficiency (http://srvgen.upct.es/efficiency.html) as described previously [61] (Additional file 1: Table S1). Primers were tested for stable, single and clear amplification products by end-point PCR with genomic DNA, visualized on 1.5% agarose gels (Additional file 1: Figure S1) and by quantitative PCR to assess the melting profile of the PCR products (Additional file 1: Figure S2).

### Quantitative PCR

Total RNA was isolated from 100 mg homogenized plant material using an RNeasy Mini Kit for leaves and pods without seeds (Qiagen, Hilden, Germany), and a phenol-based method for seeds [62]. RNA concentration and purity was estimated from the ratio of absorbance readings at 260 and 280 nm. cDNA synthesis was performed with 0.5 μg of total RNA using M-MLV reverseTranscriptase (Maxima First Strand cDNA kit for RT-qPCR, with dsDNase, ThermoFisher Scientific) according to the manufacturer instruction. Genes were amplified for three and four biological replicates from the growth chamber and field experiment, respectively, and two technical replicates in a Stratagene Mx3000P qPCR system (www.agilent.com), with sequence-specific primers (Additional file 1: Table S1) synthesized by Invitrogen (www.invitrogen.com) using SYBR-Green Mastermix (ThermoFisher Sciencific) and a 25 ng RNA equivalent of cDNA. The reaction mix was subjected to the following protocol: 95 °C for 30 s followed by 45 cycles of 95 °C for 10 s, 57 °C for 15 s and 72 °C for 15 s, and a subsequent standard dissociation protocol.

### Bioinformatics and statistical analysis

For the identification of stable reference genes during different developmental stages and tissue, PCR efficiencies and CT values were used in a web-pipeline that contains the different PCR analysis softwares Bestkeeper, Normfinder, Delta CT and Genorm. PCR efficiency was calculated as described before [61]. Data from different analysis was pooled and ranked using Rank-Aggreg (Pihur and Datta, [63]). We used the software Geomean to obtain a ranking value of the candidate reference genes [41].

Statistical analysis of diurnal gene expression profiles for clock relates genes and storage protein related genes was performed using the normalized cycle threshold (Ct) values calculated as described previously [13]. A PCR efficiency of 2 for all primer combinations was used for the calculation of normalized expression (NE) based on efficiency calculations, which were performed as described previously with the qpcR R package [61, 64]. Average efficiencies were 1.98 for *VuEF1A*, 1.99 for *VunGI,* 1.97 for *VunELF3*, 1.95 for *VunTOC1*, 1.99 for *VunLHY*, 1.99 for *VunCVC*, 1.93 for *VunLEG* and 1.99 for *VunLEGJ*. JTK-Cycle method was applied for the determination of existence of a circadian biological rhythm represented in the transcriptome data [65] using the R package “MetaCycle” that provides functions and methods (JTK_CYCLE, Lomb-Scargle and ARSER) for detecting rhythmic signals from time series datasets (https://cran.r-project.org/web/packages/MetaCycle/index.html). JTK_CYCLE results include the *P* value (Pval, significative if *P* < 0.05), period (Per), phase (Phase) and amplitude (Amp). Period is defined as the time between two consecutive peaks. Phase is considered as the time point with the peak and amplitude is the difference between the peak (or minimum) and the mean value of the wave.

Statistical analysis for gene expression was performed using group-wise comparison with the REST program [66]. Phenotypic data were analysed for homogeneity of variance with the Fligner-Killeen test in R. The parameters showing homogeneity of variance were analysed using ANOVA and Tukey’s HSD test, while the non-parametric data were analysed using Wilcoxon signed rank test with continuity correction in R version 3.2.3.

### Phylogenetics

For phylogenetic reconstructions, the identified *V. unguiculata* genes were used to identify orthologues and paralogs from other legumes. Sequences were identified by TBLASTN or BLASTP and downloaded from Phytozome [67] or NCBI. Translated cDNAs were aligned with CLUSTALW [68]. We used MEGA7 for evolutionary analysis [69]. The evolutionary history was inferred using the Neighbor-Joining method [70]. The bootstrap consensus tree inferred from 500 replicates was taken to represent the evolutionary history of the analysed taxa [71]. Branches corresponding to partitions reproduced in less than 50% bootstrap replicates are collapsed. The percentage of replicate trees in which the associated taxa clustered together in the bootstrap test (500 replicates) are shown next to the branches. The evolutionary distances were computed using the Poisson correction method [72] and are in the units of the number of amino acid substitutions per site.

## Additional files

Additional file 1: Figure S1. Amplification products of all genes applied in this study, generated by end-point PCR with genomic DNA and visualized on 1.5% agarose gels. **Figure S2.** Dissociation curve of the genes applied in this study for selected tissues. **Figure S3.** Alignments between the sequence of the identified *V. unguiculata* ESTs (NCBI and Noble VuGEA) and PCR products of each gene amplified from genomic DNA of *Vigna unguiculata*. **Table S1.** List of analysed reference genes, clock genes and protein storage genes. (DOCX 270 kb)

## Abbreviations

ACT: B-ACTIN
*ACT27*: *ACTIN 2/7*
*AtTOC1*: *Arabidopsis thaliana TIMING OF CAB EXPRESSION1*
CVC: Convicilin
*CYP*: *CYCLOPHYLIN*
*EF1A*: *ELONGATION FACTOR 1-A*
*EF1B*: *ELONGATION FACTOR 1-B*
*GI*: *GIGANTEA*
*GmCCA1*: *Glycine max CIRCADIAN CLOCK ASSOCIATED1*
*GmELF4*: *Glycine max EARLY FLOWERING4*
*GmG1*: *Glycine max GIGANTEA1*
*GmLHY*: *Glycine max LATE ELONGATED HYPOCOTYL*
*GmPRR*: *Glycine max PSEUDO RESPONSE REGULATOR*
*GmTOC1*: *Glycine max TIMING OF CAB EXPRESSION1*
LEG: Legumin
*LHY*: *LATE ELONGATED HYPOCOTYL*
*LUX*: *LUX ARRHYTHMO*
*PRR*: *PSEUDO RESPONSE REGULATOR*
*SKIP16*: *ASK-INTERACTING PROTEIN 16*
*TOC1*: *TIMING OF CAB EXPRESSION1*
*TUA4*: *ALPHA TUBULIN*
*TUB4*: *BETA TUBULIN*
*UKN2*: HYPOTHETICAL UNKNOWN PROTEIN
VIC: Vicilin
*VuEF1A*: *Vigna unguiculata ELONGATION FACTOR 1-A*
*VunCVC*: *Vigna unguiculata CONVICILIN*
*VunELF3*: *Vigna unguiculata EARLY FLOWERING3*
*VunGI*: *Vigna unguiculata GIGANTEA*
*VunLEG*: *Vigna unguiculata LEGUMIN*
*VunLEGJ*: *Vigna unguiculata LEGUMIN J*
*VunLHY*: *Vigna unguiculata LATE ELONGATED HYPOCOTYL*
*VunTOC1*: *Vigna unguiculata TIMING OF CAB EXPRESSION1*
*VuVIC*: *Vigna unguiculata VICILIN*
