# Supplementary material for: Diel pattern of circadian clock and storage protein gene expression in leaves and during seed filling in cowpea (Vigna unguiculata)
Source: BMC Plant Biol. 2018 Feb 14;18:33. doi: 10.1186/s12870-018-1244-2 (PMC5813328; doi:10.1186/s12870-018-1244-2)
Supplement: Supplementary file 1 — Amplification products of all genes applied in this study, generated by end-point PCR with genomic DNA and visualized on 1.5% agarose gels. Figure S2. Dissociation curve of the genes applied in this study for selected tissues. Figure S3. Alignments between the sequence of the identified V. unguiculata ESTs (NCBI and Noble VuGEA) and PCR products of each gene amplified from genomic DNA of Vigna unguiculata. Table S1. List of analysed reference genes, clock genes and protein storage genes. (DOCX 270 kb) [file 12870_2018_1244_MOESM1_ESM.docx]

Supplementary material

Diel pattern of circadian clock and storage protein gene expression in leaves and during seed filling in cowpea (*Vigna unguiculata*)

Julia Weiss^1^*, Marina Martos-Fuentes^1^, Lisa Letourneux^2^, Marta Isabel Terry-López^1^, Victoria Ruíz-Hernández^1^ Juan A. Fernández^3^ and Marcos Egea-Cortines^1^


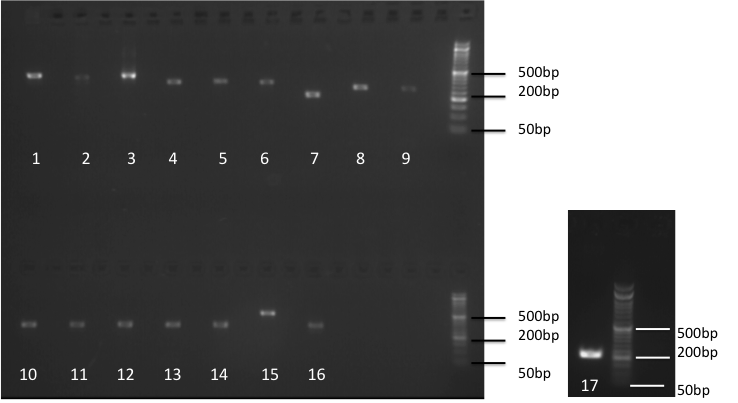


Fig. S1. (1) *CVC* (2) *ACT* (3) *VIC* (4) *CYP* (5) *EF1A* (6) *EF1B* (7) *GI* (8) *LEGJ* (9) *LEG (*10)  *LHY* (11) *SKIP16* (12) *TOC1* (13) *TUA4* (14) *TUB4* (15) *ACT27* (16) *UKN2* (17) *ELF3.*


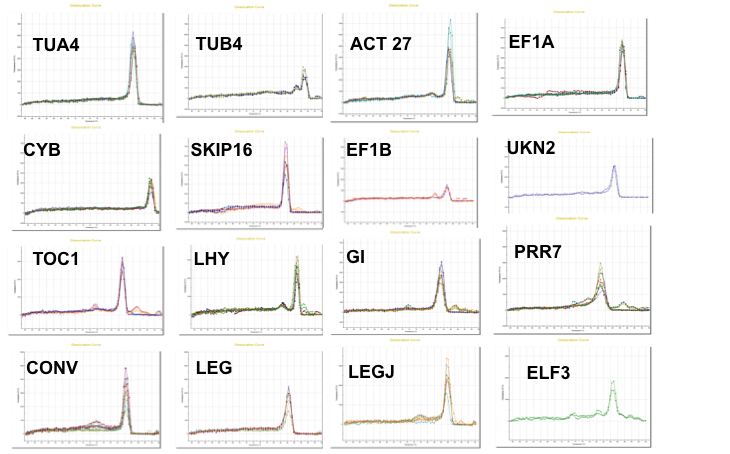


Fig. S2. Dissociation curve of the genes applied in this study for selected tissues.

VungCONV ---------------------------------------CTCAGCTCTATACCTCTGAAC

Vun_T03561.1 ATAAGTTGCTGGGATTACAAGTACATCCTCGTCAGACAACTCAGCTCTATACCTCTGAAC

*********************

VungCONV TTCCCCACTTTCCTCTTGTTGCTGTTCTCTTACTCCAACAAGCTCAGCGTTTGCTTCTCC

Vun_T03561.1 TTCCCCACTTTCCTCTTGTTGCTGTTCTCTTACTCCAACAAGCTCAGCGTTTGCTTCTCC

************************************************************

VungCONV TTCATTAACCACCAGCATGACCATAGCCTCTGAATTGTAGTGTGGTAGAAGAAGAGCTCC

Vun_T03561.1 TTCATTAACCACCAGCATGACCATAGCCTCTGAATTGTAGTGTGGTAGAAGAAGAGCTCC

************************************************************

VungB-actin NNNNTCGTCTTGATTTGGCTGGTCGTGATCTCACTGATTTTCTGATGAAGATTCTGACTG

FR839671.1 TCCTTCGTCTTGATTTGGCTGGTCGTGATCTCACTGATTTTTTGATGAAGATTCTGACTG

************************************* ******************

VungB-actin AGCGTGGATACTCTTTTACCACCTCAGCAGAGCGNGAAATTGTGAGGGATATGAAGGAGA

FR839671.1 AACGCGGATATTCTTTTACCACCTCAGCAGAGCGTGAAATTGTTAGGGATATGAAGGAAA

* ** ***** *********************** ******** ************** *

VungB-actin AGCTGGCATACATAGCCCTGGACTATGANCAGGAGCTAGAGACATCGAAGACCAGCTCTG

FR839671.1 AGCTGGCTTACATAGCCCTCGACTACGAGCAAGAGCTGGAGACATCCAAGACCAGCTCTG

******* *********** ***** ** ** ***** ******** *************

VungB-actin CAGTGGAGAAGAGCTACGAGTTGCCTGATGGGCAGGTTATCACCATTGGAGCTGAGCGTT

FR839671.1 CAGTGGAGAAGAGCTATGAGTTGCCTGATGGGCAGGTTATCACTATTGGCGCTGAGCGTT

**************** ************************** ***** **********

VungB-actin TCAGGTGTCCAGAGGTGTTGTACCAACCATCCATGGTAGGAATGGAAGCAGTAGGCAANG

FR839671.1 TCAGGTGTCCGGAGGTTTTGTACCAACCATCCATGGTAGGAATGGAAGCAGTAGGCATTC

********** ***** ****************************************

VungCyclophylin ---------------------------GCAAACTTCGAGCCGTAAATGGACTCACCTCCG

Vun_T05892.1 GTGTGCTTCTTGATGAAGTTTTCGTCCGCAAACTTCGAGCCGTAAATGGACTCACCTCCG

*********************************

VungCyclophylin GTGCCGTTTCCGGCGGTGAAGTCTCCGCCCTGGCACATGAAGTTGGGGATGACGCGGTGG

Vun_T05892.1 GTGCCGTTTCCGGCGGTGAAGTCTCCGCCCTGGCACATGAAGTTGGGGATGACGCGGTGG

************************************************************

VungCyclophylin AAAGAGGATCCCTTGTAGTGGAGGGGCTTGCCGCTGCGGCCTACTCCCTTCTCGCCGGTG

Vun_T05892.1 AAAGAGGATCCCTTGTAGTGGAGGGGCTTGCCGCTGCGGCCTACTCCCTTCTCGCCGGTG

************************************************************

VungCyclophylin CAGAGGGCGCGGAAGTTCTCGGCTGTGCGGGGAGTGGTGTCCGCGAAGAGCTCCATGACT

Vun_T05892.1 CAGAGGGCGCGGAAGTTCTCGGCTGTGCGGGGAGTGGTGTCCGCGAAGAGCTCCATGACT

************************************************************

VungCyclophylin ATGCGGCCTGCGGGTTGGCCTCCGATGGTCATGTCGAAGAAGACCTTAGGGTTGGGCATT

Vun_T05892.1 ATGCGGCCTGCGGGTTGGCCTCCGATGGTCATGTCGAAGAAGACCTTAGGGTTGGGCATT

************************************************************

VungCyclophylin GTTGCGTTGNAATTGAATTN----------------------------------------

Vun_T05892.1 GTTGCGTTGCAATTGAATTGAGAGTTGAAGGAGTGAGAGAGATCAAAGGCGAGGACTTCC

********* *********

VungEF1A -GCCTGGTATGGTGGTGACTTTTGCTCCCACTGGTTTGACAACTGAAGTTAAGTCTGTTG

Vun_T23609.1 AGCCTGGTATGGTGGTGACTTTTGCTCCCACTGGTTTGACAACTGAAGTTAAGTCTGTTG

***********************************************************

VungEF1A AGATGCACCACGAGGCTCTCCAAGAGGCTCTACCAGGTGACAATGTTGGCTTCAACGTGA

Vun_T23609.1 AGATGCACCACGAGGCTCTCCAAGAGGCTCTACCAGGTGACAATGTTGGCTTCAACGTGA

************************************************************

VungEF1A AGAACGTTGCTGTGAAGGATCTCAAGCGTGGTTTCGTTGCATCCAACTCCAAGGATGACC

Vun_T23609.1 AGAACGTTGCTGTGAAGGATCTCAAGCGTGGTTTCGTTGCATCCAACTCCAAGGATGACC

************************************************************

VungEF1A CTGCCAAGGAAGCTGCCAACTTCACATCCCAAGTCATCATCATGAACCATCCTGGCCAGA

Vun_T23609.1 CTGCCAAGGAAGCTGCCAACTTCACATCCCAAGTCATCATCATGAACCATCCTGGCCAGA

************************************************************

VungEF1A TCGGAAACGGCTATGCACCAGTGCTCGATTGCCACACTTCCCACATTGCAGTGAAGTTCG

Vun_T23609.1 TCGGAAACGGCTATGCACCAGTGCTCGATTGCCACACTTCCCACATTGCAGTGAAGTTCG

************************************************************

VungEF1B ------------------------------------TGGGTTTTCAGGGATCAGTTTACA

Vun_T21512.1 CTCAAGTCCCTCAACGACTTTCTTTCTGGAAAGACCTACGTTTCTGGGGATCAGTTTACA

* **** **************

VungEF1B AAGGACGACATCAAAGTGTACGCTGCCGTTTTGGANAAGCCGGGCGATGCTTTCCCCAGT

Vun_T21512.1 AAGGACGACATCAAAGTGTACGCTGCCGTTTTGGAGAAGCCGGGCGATGCTTTCCCCAGT

*********************************** ************************

VungEF1B GTTGGCAAGTGGTACGAGGTTGTCTCATCTAACCTTGCTGCAAGGTTTGTTCTTTTGCTC

Vun_T21512.1 GTTGGCAAGTGGTACGAGGTTGTCTCATCTAACCTTGCTGCAAGTTTCCCTGGCAATGCT

******************************************** ** *

VungGI ---------------------GCTACAGGACCTTGCTTCTTCATTTTCCTATTTTTTGAT

Vun_T01130.1 AGAACATAAGAATCAAACGCTGCTACAGGACCTTGCTTCTTCATTTTCCTATTTTTTGAT

***************************************

VungGI TCTTTCTGGTTTTCNGANGTTTGTAAGTCCTCAATGATTTCCTCAGCTGGATAGTTATCT

Vun_T01130.1 TCTTTCTGGTTTTCAGATGTTTGTAAGTCCTCAATGATTTCCTCAGCTGGATAGTTATCT

************** ** ******************************************

VungGI TCACCTCTTGGCCTCTTGCTTCCACTGAATTGAGCTTCATGACTGACGCAGACAGTTAAG

Vun_T01130.1 TCACCTCTTGGCCTCTTGCTTCCACTGAATTGAGCTTCATGACTGACGCAGACAGTTAAG

************************************************************

VungGI ACAACAAAAAGTAGTCGTGAAGCGAGCTCTACAG--------------------------

Vun_T01130.1 ACAACAAAAAGTAGTCGTGAAGCGAGCTCTACAGACGCACATGATTCTAAGAAAAGGGAA

**********************************

VungLegJ --------------------GGACAACATACTGGGCACTAAGTCCGAATTTTTTGAGAGC

Vun_T05146.1 GTAGATTCCATTCTTGTAGAGGACAACATACTGGGCACTAAGTCCGAATTTTTTGAGAGC

****************************************

VungLegJ TGGGAAACTTTGGCTGTTGAGGTTTTTGATGCGACCGGCTTTAGGGTTATAGAAGTCAGC

Vun_T05146.1 TGGGAAACTTTGGCTGTTGAGGTTTTTGATGCGACCGGCTTTAGGGTTATAGAAGTCAGC

************************************************************

VungLegJ GCCTGTAGGGCGAGCAATGTTGTGTTGAAGCTTGGAGGAGCAGATTGTTTCCTCAAGCCC

Vun_T05146.1 GCCTGTAGGGCGAGCAATGTTGTGTTGAAGCTTGGAGGAGCAGATTGTTTCCTCAAGCCC

************************************************************

VungLegJ ATTTGATCCATGGCGCTCTCTTNTCTCCCATTCTTCGTGTGCTTCTCCTCTTTCTACATC

Vun_T05146.1 ATTTGATCCATGGCGCTCTCTTGTCTCCCATTCTTCGTGTGCTTCTCCTCTTTCTACATC

********************** *************************************

VungLegJ TTCTTCTGCTGGTCTGTGTTCTTTCCAACTCTTCTCGTGGCA------------------

Vun_T05146.1 TTCTTCTGCTGGTCTGTGTTCTTTCCAACTCTTCTCGTGGCGGGTCTTGTGTCGCACTGA

*****************************************

VungLeg TACTTAGGGGTGGTGATTATAGAGAACCACTCCATTCCCTCTGGATCAGCAATTTTTGAG

Vun_T10886.2 GGATTAGGGGTGGTGATTATAGAGAACCACTCCATTCCCTCTGGATCAGCAATTTTTGAG

*********************************************************

VungLeg ACAACAAAAAACCTTGGCACAATGAACAAATTACCAGCTTTTATAGTTGTCTCCAAAACC

Vun_T10886.2 ACAACAAAAAACCTTGGCACAATGAACAAATTACCAGCTTTTATAGTTGTCTCCAAAACC

************************************************************

VungLeg CTATGACCATCAACTCCAACAACCTGAACCCGACCACTACCTNNGATGATATATGTAACC

Vun_T10886.2 CTATGACCATCAACTCCAACAACCTGAACCCGACCACTACCTCTGATGATATATGTAACC

****************************************** ****************

VungLeg TGCAAAGCAGAATCACAAGAGAATCCTGGAGAGCACATGGCTCCTCCATCC---------

Vun_T10886.2 TGCAAAGCAGAATCACAAGAGAATCCTGGAGAGCACATGGCTCCTCCATCCAACTTCACA

***************************************************

VungLHY ----------------------------------------------------TACACAAC

Vun_T17583.1 ATAACACAGCACGATCTTCTGTTCATCAATCATTTCTTCCGTATCCTCCCTTCACACAAC

*******

VungLHY ACAACCAGGACGATTACCAATCATTTCTTCACATGTCTTCCACATTTTCTAATCTTATTG

Vun_T17583.1 ACAACCAGGACGATTACCAATCATTTCTTCACATGTCTTCCACATTTTCTAATCTTATTG

************************************************************

VungLHY TCTCTACCTTGCTGCAAAACCCAGCAGCCCATGCTGCAGCAAGTTTCGCTGCTACATTTT

Vun_T17583.1 TCTCTACCTTGCTGCAAAACCCAGCAGCCCATGCTGCAGCAAGTTTCGCTGCTACATTTT

************************************************************

VungLHY GGCCCTATGCAAATCCAGAGACTTCAGCAGATTCTCCTAGGTGCTCTCAAGGAGGTTTCA

Vun_T17583.1 GGCCCTATGCAAATCCAGAGACTTCAGCAGATTCTCCTAGGTGCTCTCAAGGAGGTTTCA

************************************************************

VungLHY CATCTAGACAAATTGGTTCCCCTCCAAGCGTTGCAACTATTGCAGCTGCTACTGTAGCTG

Vun_T17583.1 CATCTAGACAAATTGGTTCCCCTCCAAGCGTTGCAACTATTGCAGCTGCTACTGTAGCTG

************************************************************

VungLHY CTGCAACTGCGTGGTGGGCAGCTCATGGACTGCTTCCTTTGTGTGA--------------

Vun_T17583.1 CTGCAACTGCGTGGTGGGCAGCTCATGGACTGCTTCCTTTGTGTGCTCCTCTTCATACTG

*********************************************

VungSkip16 -------------------------------CCACACTCCGTAGAAAATTGTTTTTCCTC

Vun_T09332.1 ACATCCAAGTTTGTTCTTGTGGCTTCTTCGTCCACACTCCGTAGAAAATTGTTTTTCCTC

*****************************

VungSkip16 AATTGTTCCAATGGCCAACTATATGTTGGAACCAATAAGCTTCGTTCTGAAAAAGACATA

Vun_T09332.1 AATTGTTCCAATGGCCAACTATATGTTGGAACCAATAAGCTTCGTTCTGAAAAAGACATA

************************************************************

VungSkip16 ATCCCTTGTGTACCTCAAGATCTGATAAGTTTACATCAGGAATCAAATGGTGAAGAGCAA

Vun_T09332.1 ATCCCTTGTGTACCTCAAGATCTGATAAGTTTACATCAGGAATCAAATGGTGAAGAGCAA

************************************************************

VungSkip16 CAAGATGCCATGCTACTGTGGTTAGAAGAACATGGTCGCCGTTTAGAACATGGCTTCATC

Vun_T09332.1 CAAGATGCCATGCTACTGTGGTTAGAAGAACATGGTCGCCGTTTAGAACATGGCTTCATC

************************************************************

VungSkip16 AAACTTTGTGAAAATGAATATGGCCGAAGCATTAATCTTTTCCCAGAGGAACCCCCTTTT

Vun_T09332.1 AAACTTTGTGAAAATGAATATGGCCGAAGCATTAATCTTTTCCCAGAGGAACCCCCTTTT

************************************************************

VungSkip16 TGTTCAACGGCTGTA---------------------------------------------

Vun_T09332.1 TGTTCAACGGCTGTTACTAATGGTGTGAAGGTTCGCTCGTCTGCATTGGTTATCCCTGAG

**************

VungToc1 ---TTACTTACCCAGCAGCAGCAGTATCCCTGATTCTTTTTCTATTGAGAGATCTTGTAC

Vun_T21607.1 AGATGACTTACCCAGCAGCAGCAGTATCCCTGATTCTTTTTCTATTGAGAGATCTTGTAC

* *******************************************************

VungToc1 TCCACCTGCATCTATGGAAGTTTNTCAGCAAAAGCATTACAGGGAAGAACATTCTCGGGG

Vun_T21607.1 TCCACCTGCATCTATGGAAGTTTCTCAGCAAAAGCATTACAGGGAAGAACATTCTCGGGG

*********************** ************************************

VungToc1 AGTAnTGCATCCAATAAATGGAAGTCATGGTTCCGATCCTGCTCAGCATGCTTATCCATA

Vun_T21607.1 AGTAGTGCATCCAATAAATGGAAGTCATGGTTCCGATCCTGCTCAGCATGCTTATCCATA

**** *******************************************************

VungToc1 TTATATTTCAAGAGTTGTTAATCATGTTATGATGCCATCnTCAGCACAAATGTATCAAAA

Vun_T21607.1 TTATATTTCAAGAGTTGTTAATCATGTTATGATGCCATCATCAGCACAAATGTATCAAAA

*************************************** ********************

VungToc1 GAATATCCAGGACCTGCAAAGTCATTCTGCACAGTACAATCGTCTTCCCCAATGTCCT--

Vun_T21607.1 GAATATCCAGGACCTGCAAAGTCATTCTGCACAGTACAATCGTCTTCCCCAATGTCCTCC

**********************************************************

VungTua4 ------------------------------------------------TTGCGCTTGGTC

Vun_T07354.1 TTGATTCCACATTTGAACCCAGTGGGGCACCAGTCAACAAATTGAATGGTGCGCTTGGTC

***********

VungTua4 TTGATAATGCCCACAGCTGCATTAACATCTTTAGGCACAACATCACCTCTGTACATGAGA

Vun_T07354.1 TTGATAATGCCCACAGCTGCATTAACATCTTTAGGCACAACATCACCTCTGTACATGAGA

************************************************************

VungTua4 CAGCAAGCCATGTATTTCCCATGGCGCGGATCACACTTGGCCATCATAGACGATGGCTCA

Vun_T07354.1 CAGCAAGCCATGTATTTCCCATGGCGCGGATCACACTTGGCCATCATAGACGATGGCTCA

************************************************************

VungTua4 AAGGCGCTGTTGGTGATTTCACCTACTGATAACTGCTCATGGTGAGCCTTCTCAGCTGAA

Vun_T07354.1 AAGGCGCTGTTGGTGATTTCACCTACTGATAACTGCTCATGGTGAGCCTTCTCAGCTGAA

************************************************************

VungTua4 ATCACGGGAGCATAAGATGAAAGCATAAAGTGGATCCTGGGGTATGGCACTAGATTGGTC

Vun_T07354.1 ATCACGGGAGCATAAGATGAAAGCATAAAGTGGATCCTGGGGTATGGCACTAGATTGGTC

************************************************************

VungTua4 TGGAATTCATTAACATCAACGTTCAATGCACCATCA------------------------

Vun_T07354.1 TGGAATTCATTAACATCAACGTTCAATGCACCATCAAACCTCAGGGAAGCAGTCAGTGAG

************************************

VungTub4 -----------CGTTAACTTCGTCTTCGGGCAGTCCGGCGCCGGTAACAACTGGGCCAAG

Vun_T13531.1 ATATTCCGGCCCGATAACTTCGTCTTCGGGCAGTCCGGCGCCGGTAACAACTGGGCCAAG

** **********************************************

VungTub4 GGTCACTACACCGAGGGTGCCGAACTTATCGATTCCGTCTTGGATGTTGTGAGGAAGGAG

Vun_T13531.1 GGTCACTACACCGAGGGTGCCGAACTTATCGATTCCGTCTTGGATGTTGTGAGGAAGGAG

************************************************************

VungTub4 GCCGAGAACTGCGATTGCATGCAAGGTATCCACAAAATTTTATTTTTTACCATATTAATT

Vun_T13531.1 GCCGAGAACTGCGATTGCATGCAAGGGTTTCAAGTGTGCCACTCTTTGGGGGGTGGAACC

************************** * ** * *** **

VungAct27 AGAAGAATGTTTACCAGTTGTACGACCACTAGCATAAAGGGAAAGCACTGCCTGGATGGC

Vun_T14730.1 GTCCAGAACAATACCAGTTGTACGACCACTAGCATAAAGGGAAAGCACTGCCTGGATGGC

* *************************************************

VungAct27 AACATACATAGCTGGAGTGTTGAAGGTCTCAAACATAATCTGTGTCATTTTCTCTCTGTT

Vun_T14730.1 AACATACATAGCTGGAGTGTTGAAGGTCTCAAACATAATCTGTGTCATTTTCTCTCTGTT

************************************************************

VungAct27 GGCCTTGGGATTGAGTGGTGCTTCTGTGAGGAGAACAGGGTGCTC---------------

Vun_T14730.1 GGCCTTGGGATTGAGTGGTGCTTCTGTGAGGAGAACAGGGTGCTCTTCTGGGGCCACACG

*********************************************

VungUKN2 TTTGTATGTACTAAGTCCACATTCTCTTCAGAGGAGGAAGTTCTTCCTGGTAGGCCTCTT

Vun_T00647.1 AATGACAAACCTGAGGATGAGTCAGAGATAGAGGAGGAAGTTCTTCCTGGTAGGCCTCTT

** ** ** * *******************************

VungUKN2 CCACCTGAATGCAATAACGAACTTCATACGGATTATGGTGGCGCCGCCGTGAGATGGGGC

Vun_T00647.1 CCACCTGAATGCAATAACGAACTTCATACGGATTATGGTGGCGCCGCCGTGAGATGGGGC

************************************************************

VungUKN2 CTTACTCATCATAAAGATAGTGCAGCCGATTGCNANNNCCCTTTCACCCGAGTGTCATGC

Vun_T00647.1 CTTACTCATCATAAAGATAGTGCAGCCGATTGCTGTAAGGCTTGCTTGGACCAGGCTAAA

*********************************

VungELF3 -------------------------------------TGTCGGTGAGAGGTTGTTTTGGA

Vun_T20640.3 GTTTCATGGTTGTGTGTTAATCTGACGTTAATTGTGCTGTCGGTGAGAGGTTGTTTTGGA

***********************

VungELF3 TTTGTGGATAAGAGAAACATGTATTGCAAAATTTGACATAAAAGGACAAGAAAGAAAGGT

Vun_T20640.3 TTTGTGGATAAGAGAAACATGTATTGCAAAATTTGACATAAAAGGACAAGAAAGAAAGGT

************************************************************

VungELF3 AAGGTATTGTATACTGTTAGGTGTAAGCTTAGAAAGTATAAGAATGAAGAGAGGGAAGGA

Vun_T20640.3 AAGGTATTGTATACTGTTAGGTGTAAGCTTAGAAAGTATAAGAATGAAGAGAGGGAAGGA

************************************************************

VungELF3 TGATGAGAAGGTGATGGGGCCAATGTTCCCTAGGCTACATGTCAATGATACAGAAAAGGG

Vun_T20640.3 TGATGAGAAGGTGATGGGGCCAATGTTCCCTAGGCTACATGTCAATGATACAGAAAAGGG

************************************************************

VungELF3 AGGACCAAGAGCA-----------------------------------------------

Vun_T20640.3 AGGACCAAGAGCACCACCTAGGAATAAGATGGCCCTCTATGAGCAGTTTAGTATTCCCTC

*************

Fig. S3. Alignments between the sequence of the identified *V. unguiculata* ESTs (NCBI and Noble VuGEA) and PCR products of each gene amplified from genomic DNA of *Vigna unguiculata*.

| Name | Short name | NCBI accession number  Query | Accession number  V. unguiculata  (Noble VuGEA) | Amplicon size EST (bp) | Forward Primer sequence | Reverse Primer sequence |
| --- | --- | --- | --- | --- | --- | --- |
| B-Actin | Act | FR839671 | FR839671 | 295 | TTCGTCTTGATTTGGCTGGT | TGCCTACTGCTTCCATTCCT |
| Actin 2/7 | Act27 | XP_006578804 | Vun_T14730.1 | 295 | GAGCACCCTGTTCTCCTCAC | TAGCCACGCTCGGTTAAGAT |
| Cyclophylin | Cyp | NP_001235223 | Vun_T05892.1 | 294 | GCAAACTTCGAGCCGTAAAT | AATTCAATTGCAACGCAACA |
| Elongation factor 1-A | EF1A | XP_003553292 | Vun_T23609.1 | 300 | GCCTGGTATGGTGGTGACTT | GCGAACTTCACTGCAATGTG |
| Elongation factor 1-B | EF1B | XP_006588020 | Vun_T21512.1 | 300 | ATCTCACCCCTTGAGCATTG | TCTGGGTTTTCAGGGATCAG |
| Alpha tubulin | Tua4 | XP_006606007 | Vun_T07354.1 | 290 | GTTTGATGGTGCATTGAACG | TGCGCTTGGTCTTGATAATG |
| Beta tubulin | Tub4 | XP_003554108 | Vun_T13531.1 | 193 | TATCAGAAGGGTTCCCATGC | TAACTTCGTCTTCGGGCAGT |
| ASK-interacting protein 16 | Skip16 | NP_001242370 | Vun_T09332.1 | 296 | ACAGCCGTTGAACAAAAAGG | GTGGCTTCTTCGTCCACACT |
| Hypothetical protein unknown | Ukn2 | XP_003528257 | Vun_T00647.1 | 297 | TGAAGAAATTGGCCTTTGGA | AGCAATCGGCTGCACTATCT |
| Convicilin | CVC | CAB82855 | Vun_T03561.1 | 295 | CTCCAACACGTTGGGAAGAT | AGCGTTGATGGCTACTGGAT |
| Legumine B | LEGB | XP_003539704 | Vun_T10886.2 | 300 | TTCCCTTGGTTGGTGATGTT | TTCCAGCCAAGTGGGTAAAT |
| Legumine J | LEGJ | XP_003520601 | Vun_T05146.1 | 264 | AGAGGACAACATACTGGGCACT | GCCACGAGAAGAGTTGGAAA |
| Gigantea | GI | [XM_014657972.1](https://www.ncbi.nlm.nih.gov/nucleotide/951024610?report=genbank&log$=nuclalign&blast_rank=4&RID=MFM0MMJ6013) | Vun_T01130.1 | 217 | GCGTCTGTAGAGCTCGCTTC | GCTGCTACAGGACCTTGCTT |
| Timing of CAB Expression1 | TOC1 | Q9LKL2 | Vun_T21607.1 | 296 | ACTTACCCAGCAGCAGCAGT | AGGAGGACATTGGGGAAGAC |
| Late Elongates Hypocotyl | LHY | Q6R0H1 | Vun_T17583.1 | 292 | ACACAACACAACCAGGACG | CACACAAAGGAAGCAGTCCA |
| Early Flowering 3 | ELF3 | ABP81864 | Vun_T20640.3 | 217 | GCTGTCGGTGAGAGGTTGTT | GCTCTTGGTCCTCCCTTTTC |

Table S1: List of analysed reference genes, clock genes and protein storage genes.
